# Loss of the candidate tumor suppressor *ZEB1* (*TCF8, ZFHX1A*) in Sézary syndrome

**DOI:** 10.1038/s41419-018-1212-7

**Published:** 2018-12-05

**Authors:** Elisabetta Caprini, Antonella Bresin, Cristina Cristofoletti, Mauro Helmer Citterich, Valeria Tocco, Enrico Scala, Alessandro Monopoli, Roberto Benucci, Maria Grazia Narducci, Giandomenico Russo

**Affiliations:** 1 Istituto Dermopatico dell’Immacolata, IDI-IRCCS, Rome, Italy; 2 Department of Paediatric Haematology and Oncology, Istituto di Ricovero e Cura a Carattere Scientifico (IRCCS) “Bambino Gesù” Children’s Hospital, Rome, Italy

## Abstract

Cutaneous T-cell lymphoma is a group of incurable extranodal non-Hodgkin lymphomas that develop from the skin-homing CD4^+^ T cell. Mycosis fungoides and Sézary syndrome are the most common histological subtypes. Although next-generation sequencing data provided significant advances in the comprehension of the genetic basis of this lymphoma, there is not uniform consensus on the identity and prevalence of putative driver genes for this heterogeneous group of tumors. Additional studies may increase the knowledge about the complex genetic etiology characterizing this lymphoma. We used SNP6 arrays and GISTIC algorithm to prioritize a list of focal somatic copy-number alterations in a dataset of multiple sequential samples from 21 Sézary syndrome patients. Our results confirmed a prevalence of significant focal deletions over amplifications: single well-known tumor suppressors, such as *TP53*, *PTEN*, and *RB1*, are targeted by these aberrations. In our cohort, *ZEB1* (*TCF8, ZFHX1A*) spans a deletion having the highest level of significance. In a larger group of 43 patients, we found that *ZEB1* is affected by deletions and somatic inactivating mutations in 46.5% of cases; also, we found potentially relevant *ZEB1* germline variants. The survival analysis shows a worse clinical course for patients with *ZEB1* biallelic inactivation. Multiple abnormal expression signatures were found associated with *ZEB1* depletion in Sézary patients we verified that *ZEB1* exerts a role in oxidative response of Sézary cells. Our data confirm the importance of deletions in the pathogenesis of cutaneous T-cell lymphoma. The characterization of *ZEB1* abnormalities in Sézary syndrome fulfils the criteria of a canonical tumor suppressor gene. Although additional confirmations are needed, our findings suggest, for the first time, that *ZEB1* germline variants might contribute to the risk of developing this disease. Also, we provide evidence that *ZEB1* activity in Sézary cells, influencing the reactive oxygen species production, affects cell viability and apoptosis.

## Introduction

Sézary syndrome (SS) is characterized by erythroderma, lymphadenopathy, and leukemic involvement of the peripheral blood. The circulating atypical lymphocytes usually are CD4^+^ T helper cells of memory phenotype^1^. The prevalence of SS is around 0.3 cases per 100,000 people and it accounts for less than 5% of all cutaneous T-cell lymphoma (CTCL). Despite numerous efforts to characterize the events of its pathogenesis, SS remains an incurable and fatal disease. Multiple genomic array-based studies have delineated a complex profile of chromosome aberrations characterizing SS genome with multiple sporadic genetic events of gains/losses in addition to recurrent abnormalities affecting mainly chromosome 8, 9, 10, and 17^2–5^. The mutational landscape of CTCL, compiled with recent reports of next-generation sequencing (NGS) data, identified a broad range of genes variously affected by somatic copy-number alterations (SCNAs) and somatic single-nucleotide variants, involved, predominantly, in the T-cell activation and apoptosis, activation of NF-kB, JAK/STAT signaling, chromatin remodeling, and DNA damage response^6–13^. Also, NGS data confirmed that a significant proportion of the oncogenic abnormalities identified in CTCL are SCNAs with an average of ~25 SCNAs observed per tumor;^6,8,12^ of these, focal deletions, resulting in frequent hemi-homozygous loss of target tumor suppressor gene loci, account for a significant proportion of the genetic aberrations, highlighting the significance of these genes as drivers of CTCL pathogenesis^6^. However, some discrepancies still exist concerning identities and frequencies of alterations found at these putative driver genes across the various studies. Particularly, some investigators reported *ZEB1* as variously affected by deletion and somatic mutation at 10p11.22 locus^2,6,9,10^, whereas others did not^7,8^. *ZEB1* is a zinc-finger-containing transcriptional repressor which regulates interleukin (IL)-stimulated cytokine signaling in normal T cells and it is essential for the correct development of T cells during hematopoiesis^14–18^. Aberrantly expressed in a variety of human solid cancers, it is assumed to foster migration, invasion, and metastasis, promoting epithelial-to-mesenchymal transition (reviewed in ref. ^19^). On the contrary, *ZEB1* is a candidate tumor suppressor gene in adult T-cell leukemia/lymphoma (ATLL), where it contributes to TGF-β1-mediated growth suppression resistance of malignant CD4^+^ T cells and *ZEB1* mutant mice frequently undergo spontaneous CD4^+^ T-cell lymphomas^20,21^. Here, we report *ZEB1* as the target of a significant focal deletion with a correspondent transcript downregulation. DNA sequence analysis identified not only somatic inactivating mutations, but also germline variants of potential clinical impact. A tumor suppressor role for *ZEB1* in SS is further supported by our survival analysis showing a worse outcome for patients carrying *ZEB1* homozygous loss. Gene set enrichment analysis (GSEA) identified, among multiple expression signatures associated with *ZEB1* absence in SS patients, the cellular response to oxidative signal. Using *ZEB1* knockout (KO) cell lines, we verified that *ZEB1* has a role in controlling intracellular reactive oxygen species (ROS) production affecting viability and apoptosis of SS cells. Although our findings require additional functional investigations, they provide the basis to understand the onco-suppressive role of *ZEB1* in CTCLs.

## Materials and methods

### Patients and cell lines

Diagnosis of SS was based on described criteria^22^. Clinical characteristics of SS patients investigated with SNP6 arrays and GISTIC analysis are shown in Table S1. Hut78 (TIB161), H9 (HTB 176), and HH (CRL2105) cell lines established from peripheral blood of CTCL patients were obtained from American Type Culture Collection (ATCC) and grown in complete RPMI 1640 with 10% FBS (Sigma-Aldrich).

### SNP6 DNA array

Tumor CD4^+^ T lymphocytes and matched granulocyte cells (normal counterpart) were isolated as previously described;^3^ genomic DNA was extracted using ArchivePure DNA Cell/Tissue Kit (5Prime, Gaithersburg, MD) following the manufacturer instructions. DNA samples were profiled on Affymetrix Genome-Wide Human SNP 6.0 Array as per the manufacturer's protocol (Affymetrix Santa Clara, CA), and the CEL files were generated using the GeneChip Command Console software 4.0 (Affymetrix). Normalization and data modeling have been performed with dChip^23^, and tumors were analyzed using paired normal samples as the reference. The calculated tumor-to-normal log2 ratio data were then exported and segmented with the Circular Binary Segmentation algorithm (CBS; α = 0.01, permutations = 10,000)^24^ via GenePattern platform at http://software.broadinstitute.org/cancer/software/genepattern/. Thresholds for copy-number change were fixed at ± 0.3 for gains/losses. We used the same segmentation algorithm applied for SNP6 arrays also for re-analyzing our previous 10 K SNP dataset^3^ to estimate the regions of copy-number changes, similar to our previous work on PTEN analysis^25^. With respect to this work, however, we have introduced additional follow-up samples/patients arrays (indicated with N superscript in Table 1) and, for those tumors with enough specimen material for new hybridizations, we have substituted the 10 K with SNP6 platform (S superscript in Table 1); finally, for more consistency of 10 K analysis results, we have eliminated SS patients with neoplastic clonal expansion equal or below 50% (i.e., SS44 and 46 of Table S1 in Cristofoletti et al.^25^). For the identification of significant regions of SCNAs, we applied the standard GISTIC (Genomic Identification of Significant Targets in Cancer) algorithm^26^ on the segmented SNP6 data using the GISTIC GenePattern module previous removal of genomic regions of structural copy-number variations identified in the Database of Genomic Variants (DGV, http://dgv.tcag.ca/dgv/app/home).

**Table 1:** Status of *ZEB1* locus 10p11.22 (chr10:31,648,147–31,856,740)

	

*FC* fold change, *UPD* uniparental disomy determined by dChip SNP software (Caprini, E et al. 2009), *Homo Del* homozygous deletion, *WT* wild type, *n.d.* not determined, *G* germline, *S* somatic

^§^Determined by Circular Segmentation Algorithm (Olshen et al.^24^):

Loss = log2 value ≤ −0.3; Homo Del = log2 value ≤ −2.0; no change = −0.3 ≤ log2 value ≤ + 0.3

Referred to the data set of Cristofoletti et al.^25^: ^S^ indicates array sample substitution, ^N^  indicates new sample array

Note: light-gray shadow includes multiple samples from the same patient. Bold font indicates *ZEB1* DNA mutation/variation

### Real-time PCR

Total RNA extraction by TRIzol reagent (Invitrogen) was performed according to the manufacturer’s instruction. Mature miR200c was analyzed using the TaqMan microRNA assay (Applied Biosystems) with RNU48 and miR16 as normalization references. *ZEB1* expression analysis was performed with GoScript Reverse Transcriptase and GoTaq qPCR master mix (Promega), using β-*actin* as normalization reference, on ABI PRISM 7000 SDS (Applied Biosystem). Relative expression was calculated using the comparative Ct method (2^–ΔΔCt^)^27^. The following primers were used: *ZEB1* forward: 5’-TGCCAACAGACCAGACAGTG-3′ reverse: 5′-CTGTCATCCTCCCAGCAGTT-3′. β*-actin* forward: 5′-GATGAGATTGGCATGGCTTT-3′ reverse: 5′-GTCACCTTCACCGTTCCAGT-3′.

### Mutation analysis

The nine coding exons of *ZEB1* gene were amplified by genomic PCR using 12 couples of primers. PCR products were then subjected to direct Sanger nucleotide sequencing. Primers’ sequence is reported in Table S2. *ZEB1* sequence variants were assessed to determine potential effects on splicing using Human Splicing Finder Version 3.1 (available in the public domain at: http://www.umd.be/HSF3/index.html). The effects of amino acid substitutions on protein function were assessed using the computational prediction scores accessible through VarSome search engine available at: https://varsome.com/.

### Survival and statistical analyses

A time-to-event analysis was performed using nonparametric Kaplan–Meier product-limit survival estimates, and differences between Kaplan–Meier survival curves were calculated using the Mantel–Haenszel log-rank test. Analyses were performed using survival curve and survival difference modules at: http://software.broadinstitute.org/cancer/software/genepattern/.

### Gene set enrichment analysis

The GSE17601 dataset published in 2009^3^ was processed and analyzed by GenePattern (http://software.broadinstitute.org/cancer/software/genepattern) using GEOImporter module (v5) and GSEA module (v17) with the Molecular Signatures Database “Hallmarks” gene set collection; briefly, this method identifies predefined database gene sets associated with phenotypic differences utilizing *t-*test statistics^28^. The default GSEA basic parameters were used with Gene Set as permutation-type option due to the small size of the dataset (<7); to find gene sets that correlate with *ZEB1* expression profile (continuous phenotype label), Pearson metric was used for ranking genes.

### CRISPR/Cas9 knockout

CRISPR–Cas9 technique was used to knock out *ZEB1*. All-in-one vectors (pCLIP-ALL-hCMV-Puro) expressing Cas9 nuclease and specific or control gRNA were purchased from Transomic Technologies (Huntsville, AL, USA). For detailed information on the construction of *ZEB1* heterozygous and homozygous knockout cell lines, please refer to Supplementary Methods. Briefly, SS cell lines were first characterized for endogenous *ZEB1* level (see Figure S1), and H9 cell line was chosen for knockout. Then, Amaxa™ Nucleofector™ Technology (Lonza Cologne GmbH) was used for cell transfection, and selection was performed with 1 µg/mL of puromycin for 72 h. Next, clonal cells were generated by single-cell dilutions and screened by a custom-designed drop-off assay performed with droplet-digital PCR (Biorad) as illustrated in Figure S2. Finally, western blot analysis was used to assess the level of ZEB1 protein as shown in Figure S3.

### Western blot

Western blot (WB) analyses were conducted as previously described^25^. Membranes were probed with primary antibodies for ZEB1 (1:400; Santa Cruz) and β-actin (1:5.000; Santa Cruz) (Santa Cruz Biotechnology, Inc., Dallas, TX, USA). Immunodetection was performed with appropriate horseradish peroxidase-linked secondary antibodies and enhanced chemiluminescence detection reagents (GEHealthcare, Amersham Biosciences, Little Chalfont, UK). The film was scanned on a GS-710 Calibrated Imaging Densitometer and analyzed by means of Quantity One Software Version 4.1.1 (Bio-Rad Laboratories, Hercules, CA, USA) and by Image J open-source software (https://imagej.net).

### ROS generation assay

Cells were stained with the oxidation-sensitive dye H2DCFDA (20 μmol/L) (Abcam) for 30 min. Then cells were treated with the indicated concentration of glucose oxidase (from *Aspergillus niger*, Sigma-Aldrich) for 4 hours, and ROS generation was determined by fluorescence-activated cell sorting (FacsCalibur, BD Biosciences, San Jose, CA, USA).

### Cell viability and cell death assays

The cells were treated with increasing concentrations of glucose oxidase, as indicated, for 24 hours and measured by the methyl thiazolyl tetrazolium (MTT) assay using ELISA reader (Biorad). Each experiment was performed three times, and results are shown as means ± SEM. Apoptosis was assessed by AnnexinV–FITC and propidium iodide (Sigma-Aldrich). Samples were acquired on a flow cytometer and analyzed using CellQuest software (BD Biosciences).

### Statistical analysis

Where not otherwise specified, statistical analyses were performed with GraphPad PRISM 6 software (GraphPad Software Inc., La Jolla, CA). Differences were evaluated with two-tailed Student’s *t* test and Pearson correlation test. *P* ≤ .05 was considered significant.

## Results

### GISTIC analysis of somatic DNA copy-number alterations in Sézary syndrome identified *ZEB1* gene as the target of a highly significant focal deletion of 10p11.22

**Fig. 1:**
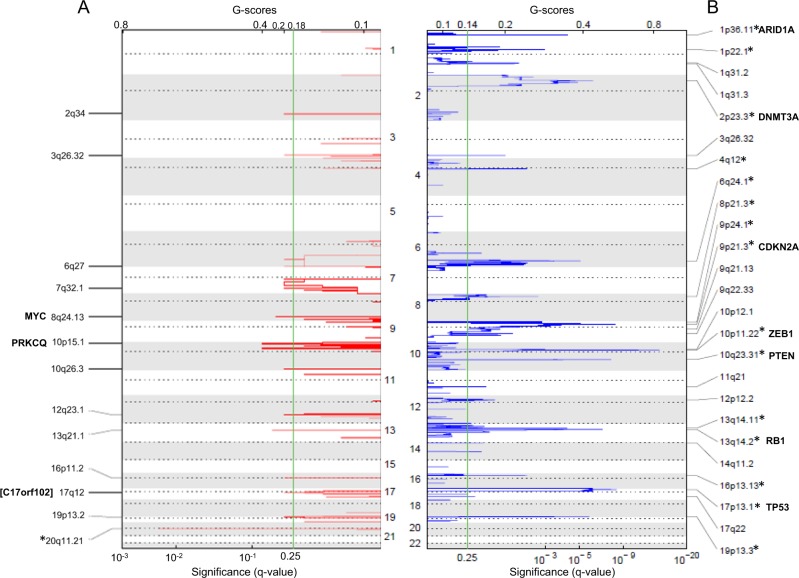
Gistic plots. Regions of gain **A** and loss **B** delineated by GISTIC analysis of 33 SS samples. Significance is reported as false discovery rate-corrected q-value. Chromosomes are indicated in the middle and the labeled cytobands correspond to the peak of significance, *regions with q-values < 0.01. Genes of interest are indicated, the gene nearest to the GISTIC peak is in brackets

We used SNP 6 arrays and GISTIC2.0 algorithm to identify genes targeted by focal SCNAs, likely representing candidate “drivers” for cancer growth^26,29^, in a dataset composed of 33 samples, collected at different time points, from 21 SS patients and 3 CTCL cell lines (Hut78, H9, HH). Applying the default threshold for significance (qv.thresh = 0.25), we found 13 regions of gains and 25 genomic losses across our dataset (Fig. 1 and Table S3). Considering as significant all events with false discovery rate (FDR) q-values < 0.01, only 20q11.21 could be taken into account among the focal amplifications; this locus, however, was found only in the Hut78 and H9 cell lines, but in none of the SS samples (data not shown), suggesting that it represents an alteration related to the in vitro replication. If the chromosomes most frequently affected by copy-number gains were considered (i.e., chromosome 10p, 8/8q, and 17q^2–5^), the following GISTIC segments may assume importance: 10p15.1 with 19% of SS tumors affected (4/21) spanning *PRKCQ* gene, 8q24.13, altering 62% of cases (13/21 and 3/3 cell lines) encompassing two genes, one of which is *MYC*, already cited as candidate gene in SS^2^, and 17q12, involving 52% of tumors (11/21 and 2/3 cell lines), where the GISTIC peak falls in close proximity of an uncharacterized open- reading frame (*C17orf102*) (Fig. 1). On the other hand, we found 15 focal deletions having FDR q-values < 0.01, four of which identified single consensus cancer drivers: 10p11.22 targeted *ZEB1* gene, which showed a deletion frequency of 47.6% (10/21 tumors and 3/3 cell lines); 17p13.1 interval, deleted in 81% of SS (17/21 and 1/3 cell line), identified *TP53*; 10q23.31 deletion fragment present in 57% of cases (12/21 and 3/3 cell lines) enclosed *PTEN*; and 13q14.2 deletion, affecting 19% of tumors (4/21 and 3/3 cell lines), involved *RB1*. The 9p21.3 GISTIC peak encompassed ten genes including *CDKN2A* as the main candidate cancer driver of this interval deleted in 33% of SS (7/21 and 3/3 cell lines) (Table S3). The high level of significance of *ZEB1* deletion in our cohort of SS cases, prompted us to re-examine the 10 K array dataset (see Materials and methods for details) and combine the 10 K and the SNP6 results to investigate the *ZEB1* locus alterations in a total of 66 specimens from 43 SS patients and 3 CTCL cell lines (Table 1). We found loss of genetic material (defined as −2.0 < Log2 values ≤ −0.3) for about 35% (15/43) of SS cases and 3 cell lines, homozygous DNA deletions (defined as Log2 value ≤ −2.0) in ~9% (4/43) of SS patients, one case (P28) of uniparental disomy (UPD, i.e., a deletion followed by a re-duplication of the remaining allele^3^), for a total of about 46.5% (20/43) of patients, and 3 cell lines affected by *ZEB1* locus abnormalities (Table 1). Eight of ten cases with one or more follow-ups (P08, P32, P38, P39, P53, P61, P64, and P68) display *ZEB1* loss in all the samples examined, whereas in two patients (P23, P63), the deletion appears from the second follow-up (median of the first observation time 4.32 months, median of follow-up observation time 29.47 months). This suggests that *ZEB1* aberrations might be related to disease onset rather than to tumor progression. Altogether, our data identified *ZEB1* as the target of a highly significant SCNA within 10p11.22 locus affecting almost half of the SS patients and the CTCL cell lines analyzed.

### *ZEB1* mRNA expression in SS tumors is dependent on copy-number status

**Fig. 2:**
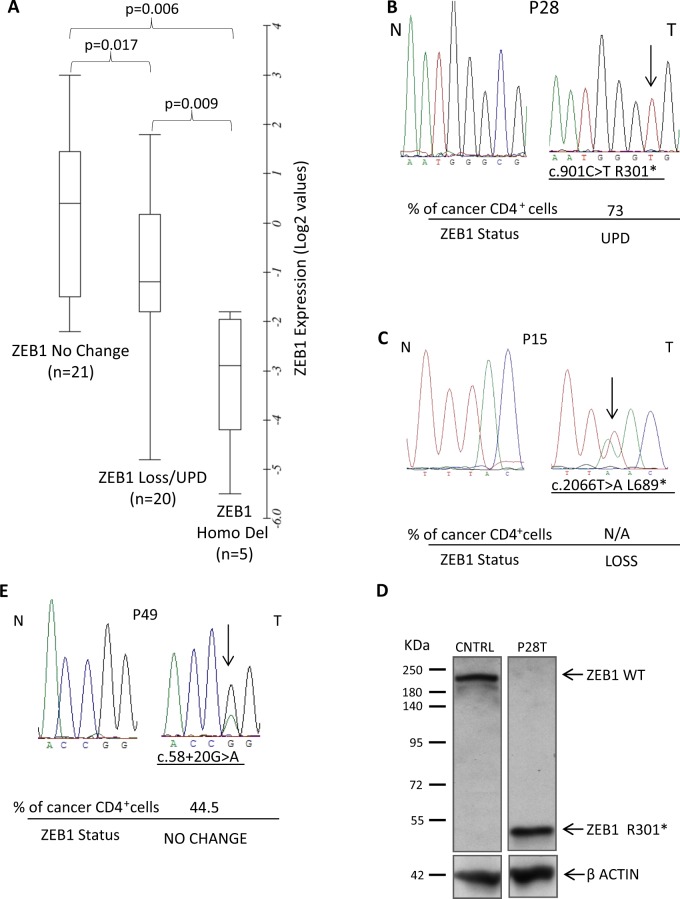
**A** Correlation analysis of *ZEB1* somatic copy-number alterations and gene expression quantitative real-time PCR data, log2 transformed; a box-plot was generated with Online Box Plot Generator (http://www.alcula.com/calculators/statistics/box-plot/) and BoxPlot Grapher (http://www.imathas.com/stattools/boxplot.html); *p*-values were calculated by two-tailed Student’s *t* test. **B** Sequence electropherograms showing the c.901 C > T, R301* somatic mutation identified in the tumor sample (T) of patient P28 compared with the normal matched DNA (N). **C** Sequence electropherograms of the *ZEB1* somatic mutation (c.2066 T > A, L689*) identified in SS patients P15. **D** Western blot analysis of ZEB1 displaying the R301* truncated protein expression in neoplastic lymphocytes of P28 compared to ZEB1 expression in the CD4 + lymphocytes of a healthy volunteer. **E** Sequence electropherogram identifying the c.58 + 20 G > A substitution in the tumor DNA (T) of patient P49 compared with the normal matched DNA (N). For each SS patient sequenced, the percentage of CD4 + neoplastic cells and the *ZEB1* status is indicated; a cutaneous biopsy of P15 has been used for sequence analysis of tumor DNA (T) since the patient underwent bone marrow transplantation

To verify whether the high frequency of *ZEB1* SCNA led also to an altered transcript level, we analyzed the mRNA expression in 43 samples, with available RNA, from 35 SS patients and 3 cell lines (Table 1); we used CD4^+^ T cells from four healthy controls to calibrate the fold-change (FC) values of *ZEB1* in quantitative PCR assays. Comparing samples showing loss (20/46) or homozygous deletion (5/46) with samples exhibiting no change of *ZEB1* copy number (21/46), we found *ZEB1* expression values proportional to copy-number data with significant statistical differences (Fig. 2A), indicating that *ZEB1* mRNA level correlates with its genomic locus status.

### *ZEB1* mutation analysis in SS patients

**Fig. 3:**
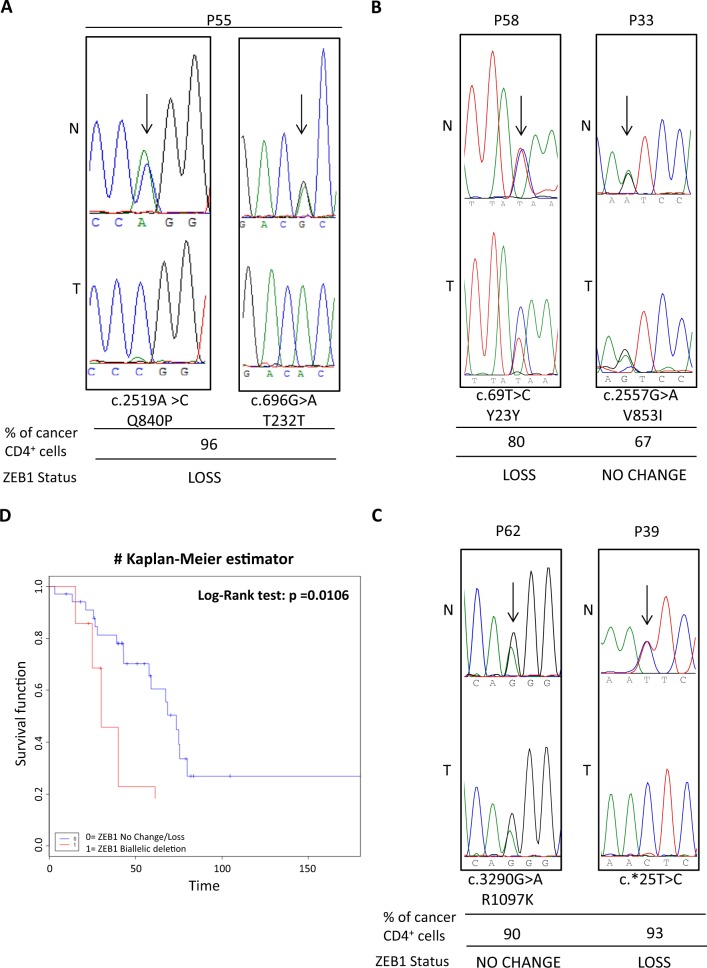
**A** Sequence electropherograms showing the germline heterozygous c.2519 A > C (Q840P) (left) and c.696 G > A (T232T) (right) rare variations identified in the normal DNA of P55 (N) which become hemizygous following the loss of the wild-type allele in the tumor sample (T). **B**, **C** Sequence electropherograms of DNA patients displaying rare c.69 T > C, Y23Y, and the newly identified c.2557 G > A, V853I; c.3290 G > A, R1097K; c.*25 T > C germline variants. The percentage of neoplastic CD4 + cells are indicated for each patient as well as the *ZEB1* status. **D** Comparison of survival time (months) between SS patients carrying no change or loss of copy number (*n* = 34, blue line) vs. biallelic deletion (*n* = 7, red line) of *ZEB1* gene. Patients with truncating (R301*, L689*) or loss-of-function mutations (Q840P) associated with loss were included in the biallelic deleted group. Forty-one out of 43 patients were used for this analysis because one was lost at follow-up and the other had an evolution of SS after a long stable MF disease

We then performed *ZEB1* DNA sequence analysis in 38 SS patients and 3 CTCL cell lines. We detected seven coding nucleotide substitutions in 6/38 cases and four noncoding variations affecting 14 patients and 1 cell line. The available normal matched DNA allowed us to establish that three were somatic and eight were germline substitutions (Table 1). Two somatic mutations were nonsense predicting to generate *ZEB1*-truncated proteins: c.901 C > T (R301*) in P28 and c.2066 T > A (L689*) in P15 (Fig. 2B, C). These SS cases display also UPD and loss of *ZEB1* locus, respectively. Western blot analysis revealed that c.901 C > T mutated gene produces a single fragment of a lower molecular mass compared with the full-length protein (Fig. 2D); if, similarly, the c.2066 T > A mutated gene is translated in a shortened 689 amino acids, then both proteins will likely result in defective molecules (Fig. S4). The third somatic variation, identified in P49 associated with a normal copy number of *ZEB1*, is a transition (c.58 + 20 G > A) occurring within the first intron of the gene (Fig. 2E). Computational analysis predicted the nucleotide change as probably having no effect on splicing (see Materials and methods for mutation analyses). Since the eight other substitutions were germline, we sought to determine their pathogenicity using data from dbSNP and Genome Aggregation Database (gnomAD)^30^ (Table 2). The intronic substitutions c.481 + 64 A > G and c.685–15 G > A, identified in 10/38 and 2/38 SS patients, respectively, are genetic variants reported as having minor allele frequencies (MAF) in dbSNP, or allele frequencies of the alternative (non-reference) allele (AF) in gnomAD, above 0.05, thereby likely representing a genetic variant of benign nature. Conversely, we found three rare genetic variations (MAF and AF < 0.05): one missense transversion (c.2519 A > C, Q840P) and two synonymous transitions (c.696 G > A, T232T and c.69 T > C,Y23Y). The missense substitution is reported as having clinical relevance owing to its association with late-onset Fuchs corneal dystrophy (FCD) and shown to cause loss of function (LoF) by an in vivo complementation assay^31^. Noticeably, the patient (P55) carrying this variant displays also concomitant loss of *ZEB1* locus (Fig. 3A, left panel) possibly resulting in the deficiency of a functional allele. The c.696 G > A, T232T was found in the same patient (P55) (Fig. 3A, right panel), and the c.69 T > C,Y23Y in P58 SS case (Fig. 3B, left panel) both combined with *ZEB1* deletions; these silent rare variations were tested for their effects on putative exonic splicing enhancers and silencers^32^, but the analysis did not detect any significant splicing motif alteration; it cannot be excluded, however, that they might have other consequences on mRNA stability and/or translation efficiency^33^. Finally, we identified the following three germline variants: (1) the c.2557 G > A (V853I) missense substitution in P33 associated with no change of *ZEB1* locus (Fig. 3B, right panel), (2) the c.3290 G > A (R1097K), found in P62 carrying a normal *ZEB1* copy number, and (3) c.*25 T > C present in P39 with *ZEB1* loss (Fig. 3C), none of which was found in the dbSNP and gnomAD (Table 2). It is worth noting that these variations were all absent also from the COSMIC (Catalogue of Somatic Mutations in Cancer) database, suggesting that they do not occur in sporadic cancers. Multiple lines of computational evidence suggest no impact of c.2557 G > A (V853I) and c.3290 G > A (R1097K) on gene or gene product; however, due to the absence from a large general population, the significance of these variant alleles remains uncertain.

**Table 2:** *ZEB1* germline substitutions

					Alternative allele frequencies (AF)	
Nucleotide position^$^	Amino acid change	Exon/intron	No. of cases (%)	dbSNP rs* global MAF	gnomAD^§^ exome	gnomAD^§^ whole genome
c.69 T > C	Y23Y	Exon 2	1/38 (2.63)	rs775270565 C = 0.00002/2	C = 0.00001223	C = 0.0000323
c.481 + 64 A > G	–	Intron 4	10/38 (26.3)	rs2839664 G = 0.0787/394	–	G = 0.1308
c.685-15 G > A	–	Intron 5	2/38 (5.2)	rs220060 G = 0.0787/394	A = 0.9458	A = 0.9197
c.696 G > A	T232T	Exon 6	1/38 (2.63)	rs149166539 A = 0.0002/1	A = 0.001227	A = 0.0009054
c.2519 A > C	Q840P	Exon 7	1/38 (2.63)	rs118020901 C = 0.0030/15	C = 0.007583	C = 0.009168
c.2557 G > A	V853I	Exon 7	1/38 (2.63)	Absent	Allele not found despite > 20X coverage	Allele not found despite > 20X coverage
c.3290 G > A	R1097K	Exon 9	1/38 (2.63)	Absent	0.00	Allele not found despite > 20X coverage
c.*25 T > C	–	Exon 9	1/38 (2.63)	Absent	Allele not found despite > 20X coverage	Allele not found despite > 20X coverage

^$^Ref Seq NM_030751 (CDS), from 5′to 3′ end

* https://www.ncbi.nlm.nih.gov/snp (Build 151). The current default global population is 1000Genome phase 3 genotype data from 2500 worldwide individuals, released in the May 2013 dataset. ^§^Genome aggregation database r2.0.2 accessed through https://varsome.com/. The data set spans 123,136 exome sequencesand 15,496 whole-genome sequences from unrelated individuals

### Biallelic inactivation of *ZEB1* reduces survival in SS patients

We then evaluated if *ZEB1* deficiency might affect SS patients survival. In our series, median overall survival was 48 months for *ZEB1* no change or loss of copy number (*n* = 34), compared with 30 months for cases with biallelic loss (*n* = 7), including patients carrying inactivating (i.e., nonsense mutations) or Q840P LoF variation associated with loss. Overall survival analysis with the Kaplan–Meier estimator revealed reduced survival probability among patients with biallelic loss (log-rank test, *P* = 0.0106; Fig. 3D). Thus, beyond representing a potential oncogenetic event, lack of *ZEB1* function also affects SS prognosis.

### Gene sets enriched in *ZEB1*-depleted SS samples

To gain further insights into *ZEB1* role in SS disease, we used our previously published gene expression (GE) dataset (GSE 17601^3^) to explore the transcriptional patterns of *ZEB1*-deficient and *ZEB1*-expressing patients. Considering the available 32 arrays in the GE dataset, we had corresponding *ZEB1* genetic and expression data for 19 samples (Table 1: P02T, P05T, P08T2, P22T, P23T3, P25T, P28T, P30T1, P32T1, P33T, P34T, P35T, P36T, P37T, P38T1, P39T1, P40T3, P43T2, P45T1). Clustering these SS cases for *ZEB1* expression, we had six samples showing a positive log2 FC values (P30T1, P33T, P35T, P36T, P39T1, P45T1) that we labeled as *ZEB1* OVER class; in order to maximize the transcriptional differences, we compared this group of cases with a comparable size set of six *ZEB1*-depleted samples, i.e., exhibiting either homozygous deletion of *ZEB1* (P05T, P22T, P32T1), UPD (P28T) or strong mRNA down-regulation with log2 FC expression level ≤ −2.0 (P23T3, P25T); for simplicity, we indicated this class as *ZEB1* HD (i.e., HomoDel). We used GSEA and the Molecular Signatures Database “Hallmarks” gene set collection^34^ to investigate the molecular alterations enriched in the two different groups of patients; The analysis yielded 32 gene sets that were enriched, with FDR < 0.25, in the *ZEB1* HD class (Table 3A). To further verify our findings and select the Hallmarks signatures dependant on *ZEB1* expression, the same dataset of samples was used to identify lists of enriched gene sets negatively correlated with the expression profile of *ZEB1* (Affymetrix ID: 210875_s_at), i.e., gene sets made up of core enriched genes mostly upregulated in *ZEB1*-deficient samples, but downregulated in *ZEB1*-expressing cases. A negative association was found for 11 gene sets (FDR < 0.25) all of which were included in the *ZEB1* HD class of enriched signatures (Table 3B). Notably, the presence of the Hallmark of IL2_STAT5 signaling either in positive association with *ZEB1* HD class or in negative correlation with *ZEB1* expression support our analyses since ZEB1 is a known repressor of IL2 transcription in T cells^14,15^. The results identified the cell response to interferon (IFN)α and IFNγ as highly significant signatures associated with *ZEB1* depletion, suggesting it might have a role in the immune-regulatory activities mediated by these cytokines in SS T lymphocytes. Of note also the correlation with the tumor necrosis factor (TNF)α signaling via NF-kB, one of the most important activated pathway conveying persistent survival signals in SS^35^. Additionally, we found enriched gene sets, such as DNA repair and the P53 pathway, indicating a potential ZEB1 control also in the regulation of genomic DNA integrity. To test this hypothesis, we compared the fraction of tumor genome affected by SCNAs for nine SS samples with *ZEB1* homo-deletions or strong mRNA under expression and eight SS tumors with *ZEB1*-positive expression calculated as the proportion of genome altered (PGA) described by Thu KL et al.^36^ No significant results were found considering either all lesions (i.e., gains and losses) or losses and gains taken separately (Supplementary Figure S5), thus, *ZEB1* depletion does not seem to increase genetic instability in SS. The more widespread changes in the transcriptional program observed comparing *ZEB1* HD with *ZEB1* OVER classes might be explained, at least in part, as the downstream effects of *ZEB1* lack of activities distinguishing the two groups. In support of this data interpretation, we found the upregulation of the oxidative phosphorylation enzymes in both types of analyses (Tables 3A and 3B), and the hallmarks of reactive oxygen species pathways and peroxisome associated with *ZEB1* HD class (Table 3A). This is of particular interest as *ZEB1* is indeed involved in ROS-induced cellular response as a crucial target of the miR-200 family members that are upregulated under oxidative stress conditions^37–41^.

**Table 3:** Gene sets enriched in *ZEB1* HD class (A). Gene sets enriched in negative correlation with *ZEB1* profile (B)

	NAME	SIZE	ES	NES	NOM p-val	FDR q-val
**(A)**						
1	**HALLMARK_OXIDATIVE_PHOSPHORYLATION**	188	0.67	2.67	0	0
2	**HALLMARK_INTERFERON_ALPHA_RESPONSE**	77	0.74	2.62	0	0
3	**HALLMARK_INTERFERON_GAMMA_RESPONSE**	171	0.65	2.58	0	0
4	**HALLMARK_COMPLEMENT**	181	0.58	2.31	0	0
5	HALLMARK_MYC_TARGETS_V1	171	0.57	2.29	0	0
6	**HALLMARK_ADIPOGENESIS**	169	0.53	2.08	0	0
7	HALLMARK_ALLOGRAFT_REJECTION	191	0.52	2.07	0	0
8	HALLMARK_REACTIVE_OXIGEN_SPECIES_PATHWAY	43	0.58	1.89	0	0.001
9	**HALLMARK_APOPTOSIS**	157	0.48	1.88	0	0.001
10	HALLMARK_IL6_JAK_STAT3_SIGNALING	85	0.51	1.85	0	0.001
11	HALLMARK_PROTEIN_SECRETION	88	0.5	1.83	0	0.001
12	**HALLMARK_IL2_STAT5_SIGNALING**	169	0.45	1.78	0	0.003
13	HALLMARK_MTORC1_SIGNALING	182	0.45	1.75	0	0.003
14	HALLMARK_FATTY_ACID_METABOLISM	140	0.45	1.73	0.001	0.004
15	**HALLMARK_DNA_REPAIR**	135	0.45	1.71	0.001	0.005
16	HALLMARK_KRAS_SIGNALING_UP	191	0.42	1.69	0	0.006
17	HALLMARK_XENOBIOTIC_METABOLISM	185	0.42	1.67	0.001	0.008
18	HALLMARK_INFLAMMATORY_RESPONSE	185	0.41	1.64	0	0.01
19	HALLMARK_HEME_METABOLISM	183	0.41	1.63	0.001	0.011
20	**HALLMARK_TNFA_SIGNALING_VIA_NFKB**	185	0.41	1.63	0.002	0.01
21	HALLMARK_G2M_CHECKPOINT	176	0.41	1.62	0.001	0.01
22	HALLMARK_UNFOLDED_PROTEIN_RESPONSE	104	0.42	1.55	0.011	0.018
23	HALLMARK_E2F_TARGETS	167	0.39	1.53	0.003	0.02
24	HALLMARK_COAGULATION	132	0.39	1.51	0.011	0.024
25	HALLMARK_CHOLESTEROL_HOMEOSTASIS	60	0.44	1.51	0.031	0.023
26	HALLMARK_PEROXISOME	94	0.4	1.45	0.041	0.04
27	HALLMARK_TGF_BETA_SIGNALING	49	0.43	1.45	0.043	0.041
28	**HALLMARK_P53_PATHWAY**	182	0.36	1.4	0.024	0.059
29	HALLMARK_ANDROGEN_RESPONSE	94	0.36	1.34	0.077	0.098
30	**HALLMARK_UV_RESPONSE_UP**	150	0.34	1.33	0.051	0.099
31	HALLMARK_ESTROGEN_RESPONSE_LATE	193	0.33	1.31	0.061	0.115
32	HALLMARK_PANCREAS_BETA_CELLS	36	0.42	1.3	0.129	0.114
**(B)**						
1	**HALLMARK_INTERFERON_ALPHA_RESPONSE**	77	−0.57	−2.86	0	0
2	**HALLMARK_INTERFERON_GAMMA_RESPONSE**	171	−0.47	−2.68	0	0
3	**HALLMARK_TNFA_SIGNALING_VIA_NFKB**	185	−0.28	−1.65	0	0.029
4	**HALLMARK_IL2_STAT5_SIGNALING**	169	−0.28	−1.6	0	0.034
5	**HALLMARK_APOPTOSIS**	157	−0.28	−1.58	0	0.031
6	**HALLMARK_DNA_REPAIR**	135	−0.28	−1.56	0.008	0.029
7	**HALLMARK_P53_PATHWAY**	182	−0.25	−1.45	0.005	0.059
8	**HALLMARK_ADIPOGENESIS**	169	−0.24	−1.39	0.014	0.081
9	**HALLMARK_OXIDATIVE_PHOSPHORYLATION**	188	−0.24	−1.37	0.02	0.089
10	**HALLMARK_UV_RESPONSE_UP**	150	−0.23	−1.28	0.059	0.153
11	**HALLMARK_COMPLEMENT**	181	−0.21	−1.21	0.073	0.239

*SIZE* size of gene set, *ES* enriched score, NES normalized enriched score, *NOM* p-val nominal p-value, *FDR q-val*, false discovery rate q-value

Bold font denotes common gene sets enriched in both analyses

### *ZEB1* is not regulated by miR200c in SS

We tried to assess if any relationship existed between *ZEB1* and miR200 expression in SS. From microarray data of our previous study on miRNA transcription profiling in SS^42^, we identify miR200c as the family member having the highest variance in expression between patients and controls; thus we performed a real time quantitative assay to estimate miR200c FC values in 18 SS patients/cell lines (P25T, P30T1, P37T, P39T1, P39T2, P40T3, P43T3, P45T1, P48T1, P51T1, P62T1, P63T1, P67T1, P68T1, P69T, HUT78, H9, and HH cell lines) using the same set of healthy donors employed for *ZEB1* expression analysis. No significant correlation emerged between the log2 FC values of *ZEB1* and miR200c in a Pearson’s correlation analysis (R = 0.0144, *p*-value = 0.956). Also, dividing the same group of samples for *ZEB1* expression level, we calibrated the miR200c FC values of the under-expressing cases (P25T, P37T, P40T3, P48T1, P62T1, P67T1, P68T1, P69T, HUT78) on the average value of the over-expressing tumors (P30T1, P39T1, P39T2, P43T3, P45T1, P51T1, P63T1, H9, HH); in this case a weak negative correlation emerged that, however, did not reach the significance (Pearson’s correlation R = −0.426, *p*-value = 0.25). Our results suggest that *ZEB1* role in oxidative phosphorylation response of SS cells is independent from miR200c expression.

### Oxidative stress and *ZEB1* depletion in SS cells

**Fig. 4:**
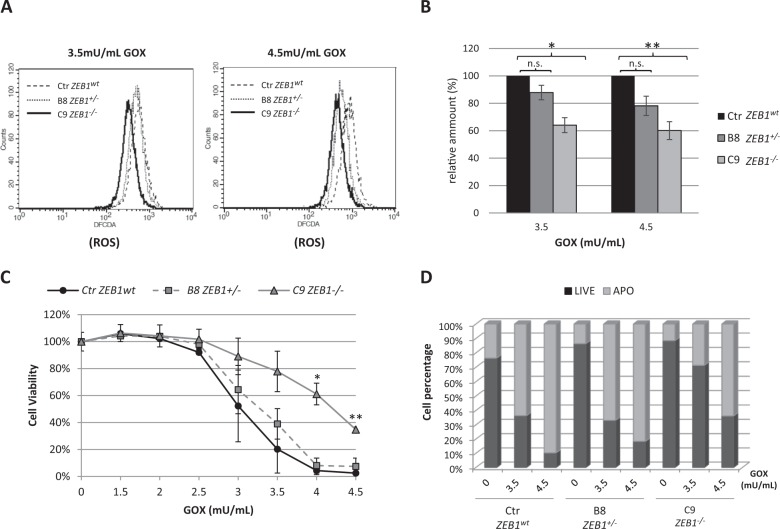
**A** Flow cytometry analysis showing the levels of ROS produced in *ZEB1*^wt^ control cells and *ZEB1* KO clones following exposure to 3.5 mU/mL and 4.5 mU/mL of GOX concentration; ROS are detected as H_2_DCFDA fluorogenic dye intensity. **B** The amount of ROS produced in *ZEB1* KO clones, relative to the WT control, following 3.5 and 4.5 mU/mL of GOX treatment for 4 h. Data are expressed as means ± SEM (*n* = 3), *p < 0.05 **p < 0.01 vs. control. **C** MTT assay showing the differences in cell viability between the *ZEB1*^wt^ control cells and *ZEB1* KO clones treated with increasing concentration of GOX; *p < 0.05, **p < 0.001 vs. control. **D** Bar graph quantifying the percentage of dead and living cell sub-populations in *ZEB1*^wt^ control and KO clones according to the indicated treatments. Data are expressed as percentage of total cell numbers. Light-gray  bars indicate Annexin V-positive apoptotic cells

The stimulation of T-cell receptor (TCR) generates an oxidative signal that is crucial to trigger the activation-induced cell death (AICD), a key process required to keep in balance the immune system^43,44^. This prompted us to study the effects of *ZEB1* deficiency in SS context of oxidative condition. To this end, we used heterozygous and homozygous CRISPR/Cas9-generated *ZEB1* KO cell clones, derived from H9 SS cell line (see Supplementary Methods), treated with glucose oxidase (GOX) which reacts with glucose and oxygen for a continuous enzymatic generation of H_2_O_2_. This reagent, used at relatively low concentration (2–5 mU/mL), it is used to reproduce a physiological condition of constant low level H_2_O_2_-mediated oxidative signal^45^. Flow cytometry analysis of cells cultured with increasing concentrations of GOX for 4 hours showed a sustained increase in intracellular ROS production, however, if compared with the WT CRISPR/Cas9 control cells (Ctr *ZEB1*^wt^), *ZEB1*^−/−^ cell clone (C9 *ZEB1*^−/−^) showed an appreciable lower amount of ROS at 3.5 and 4.5 mU/mL of GOX concentration, while the fluorescence intensity of *ZEB1*^+/−^ cells (B8 *ZEB1*^+/−^) overlapped the WT control at 3.5 mU/mL and exhibited an intermediate profile between the two genotypes at 4.5 mU/mL of GOX (Fig. 4A). When ROS levels were calculated relatively to *ZEB1*^wt^ control cells, significant statistical decreases were measured for *ZEB1*^−/−^ cells at both GOX concentrations, whereas no significant differences emerged in *ZEB1*^+/−^ clone (Fig. 4B). These results suggest that *ZEB1*-deficient SS cells are more competent to respond to H_2_O_2_-induced oxidative stress counterbalancing increasing intracellular ROS production. Cell viability assays of *ZEB1*^wt^ and KO clones showed that *ZEB1*^−/−^ cells exhibited higher cell viability with respect to *ZEB1*^wt^ and *ZEB1*^+/–^ following the exposure to increase GOX-induced oxidative stress with significant differences observed at 4.0 and 4.5 mU/mL (Fig. 4C). Flow cytometry, used to quantify cellular apoptosis in response to 3.5 and 4.5 mU/mL of GOX, revealed that *ZEB1*^−/−^ KO showed 29 and 64% of apoptotic cells, respectively; conversely, *ZEB1*^wt^ exhibited 64 and 94% of apoptosis at the corresponding GOX concentrations, comparable with the 67 and 82% values observed for *ZEB1*^+/−^ KO (Fig. 4D). Collectively, our data show that *ZEB1*-devoid SS cells are able to respond to oxidative stress counteracting ROS imbalance, this ability influences cell survival and apoptosis and it requires the absence of both *ZEB1* alleles.

## Discussion

We have generated a high-resolution SNP6 data set of SS tumors; in the absence of hallmarks of disease progression, we used multiple follow-up samples to increase the detection of positively selected clonal DNA aberration in the disease course. The GISTIC algorithm delineated a profile of significant regions of gain and loss, with a large predominance of focal deletions over amplifications. This is in line with previously published studies and support the hypothesis that deletion events, targeting critical tumor suppressors, represent a mechanism of CTCL pathogenesis^5,6^. We narrowed some of the most significant focal deletions to critical tumor suppressor targets, such as *TP53*, *PTEN,* and *RB1* (Fig. 1), thereby involving their loss as driver events in SS tumorigenesis. Recent studies, integrating copy-number and single-nucleotide variant analyses, identified these genes with variable frequencies in CTCL cohorts: *TP53* was affected in 21–92.5% of cases, *PTEN* ranged from 20 to 30%, and *RB1* and *CDKN2A* were targeted in 16–39% and 32.5–58% of tumors, respectively^6,8,9,11,46^. Our data also confirmed targets such as *PRKCQ* found altered in 20–30% of CTCLs^6,11^, and support other candidates like the chromatin-modifying genes *ARID1A*, implicated in 33–62.5% of CTCLs, and *DNMT3A* involved in 9–42.5% of cases^6,8,9,11,12^. This great variability in the alteration frequencies might depend on sample sizes/compositions and/or diversities of technical/analytical approaches. In our cohort, the GISTIC analysis identified 10p11.22 as the region most significantly affected by loss of genetic material and *ZEB1* as the target of this region; also, we found the presence of nonsense somatic mutations, predicting to generate defective proteins coupled with *ZEB1* deletion. A combined burden of deletions and somatic mutations, affecting *ZEB1*, has been reported with frequencies ranging from 45 to 65% of CTCLs^2,6,9,10^. Thus, our and previous findings support *ZEB1* as a strong candidate tumor suppressor gene in CTCL as already described in ATLL;^20^ differently from canonical cancer genes like *TP53*, *PTEN*, *RB1,* and *CDKN2A* recurrently inactivated also in other cancer types, *ZEB1* seems to be a lineage-specific tumor suppressor. Here, we show, for the first time, that SS patients harbor not only *ZEB1* somatic mutations, but also germline variations; we found that 8/11 allelic variants were of germline origin (Table 1 and Table 2). We found substitutions reported as either rare variants (MAF/AF < 5%) or totally absent from dbSNPs or gnomAD, representing, thereby, novel sequence variations (Table 2). Importantly, the Q840P (c.2519 A > C) is a *ZEB1* LoF variation associated with a rare inherited disorder of the corneal endothelium^31^. Interestingly, Hidaka et al.^20^, reported the identification of *ZEB1* c.232 A > C (N78T) variation in the Hut102 CTCL cell line, which, also, is recurrent in FCD patients and causes a protein impairment^31^. Although the germline origin of the N78T variation cannot be proved for Hut102, it is worth noting that it is absent from the COSMIC database, whereas it is cataloged as a variation with a global MAF < 5% in the dbSNP (rs80194531) and gnomAD, further supporting our findings. Rare genetic variations play an important role in human disorders, as they may cause many Mendelian and rare forms of common diseases^47^. Evolutionary theory predicts that disease alleles are likely to be rare as a result of purifying selection^47,48^ and indeed, LoF variants are especially rare^49,50^. Also, empirical evidences have shown that low frequency and rare variants are associated with complex diseases, including cancer^51–53^. Moreover, emerging evidence suggests that synonymous SNPs could affect, besides mRNA splicing, also mRNA stability, protein expression, and enzymatic activity contributing to human disease risk and other complex traits (reviewed in ref. ^54^). Although further observations and experimental validations are necessary to establish the functional consequences of *ZEB1* germline variations in SS, this is the first report to suggest a genetic risk for this rare neoplasm. Overall survival analysis, in our SS series, highlighted an association of poorer outcome with biallelic loss of *ZEB1*, which supports its recessive nature of tumor suppressor and underlines its potential role as a risk-stratification and prognostic marker. In CTCL, ZEB1 acts as a transcriptional repressor contributing to an oncogenic regulatory mechanism involving IL15, HDAC1, HDAC6, and miR-21^16^. Here, we found that *ZEB1* absence is associated with multiple dysregulated pathways (Table 3), among which, the upregulation of IL2 signaling and the oxidative stress cell response were already shown to depend on ZEB1 expression. In oxidative stress condition, *ZEB1* downregulation by miR-200c has been described; we could not confirm this relationship in SS, rather *ZEB1* expression is mainly dependant on the integrity of its genomic locus (Fig. 2A). We verified that *ZEB1*^−/−^ SS cells respond to oxidative stress with significant lower intracellular ROS levels, higher cell viability, and resistance to apoptosis. ROS mediate apoptosis in a number of cell types; in T cells, re-stimulation of previously activated T cells via the TCR increases ROS concentrations leading to AICD, an important regulatory mechanism of T-cell homeostasis^55^. Indeed, CTCLs have been shown to be AICD-resistant, while inhibition of the NF-kB pathway induces CTCL cell death via free intracellular iron and massive ROS production^56,57^. NF-kB exerts a negative control on ROS formation, which is central for the suppression of TNFα-induced apoptosis^58–61^. Interestingly, we found that upregulation of TNFα signaling via NF-kB is significantly correlated with *ZEB1* absence, suggesting a control of this pathway in SS disease. Recently, Morel et al. reported that *ZEB1* reduces cellular ROS in mammary stem cells following an oncogenic stimulus, while the expression of several detoxifying enzymes was increased; among them, they identified *MSRB3* as a transcriptional target of *ZEB1*, implying its direct role in the control of ROS production^62^. We did not find *MSRB3*, but *HIF1A*, another antioxidant gene found by Morel et al., positively correlated with *ZEB1* depletion. It would be interesting to further investigate if *ZEB1* is capable, not only to induce ROS-scavenging factors, but also to repress them in a cell-type-specific manner. Although we found molecular pathways of genomic instability associated with *ZEB1* absence, no significant differences emerged in our PGA analysis between *ZEB1*-depleted and *ZEB1*-expressing tumors; this might be due to additional independent factors contributing to DNA damage such as defects in genes deputed to genome maintenance^11^. Also, it has been shown very recently that *ZEB1* mediates the epigenetic silencing of protective type III IFN response in airway mucosal epithelium^63^. Here, we found *ZEB1* depletion highly correlated with IFNα and IFNγ response (Table 3). IFNs have been proposed to play an important role in regulating the abundance and persistence of memory T cell;^64,65^ particularly, previous studies showed that IFNα/β inhibited activated T-cell apoptosis directly, without induction of secondary mediators, promoting, among the diverse survival pathways, also the upregulation of intracellular antioxidant molecule glutathione^66–68^. Although our observations await further confirmations and mechanistic experimental validations, they provide evidence for uncharacterized *ZEB1* roles in regulating T-cell homeostasis, paving the way for future developments and treatment opportunities.

### Ethics approval and consent to participate

This study was conducted in accordance with Good Clinical Practice Guidelines and the Declaration of Helsinki and approved by the Ethical Committee of the Istituto Dermopatico dell’Immacolata (ID n. 4/CE/2015). A written consent was obtained from all patients.

## Electronic supplementary material

Table S1

Table S2

Supplementary methods

Figure S1

Figure S2

Figure S3

Table S3

Figure S4

Figure S5

List of supplementary information
