# Supplementary material for: Loss of the candidate tumor suppressor ZEB1 (TCF8, ZFHX1A) in Sézary syndrome
Source: Cell Death Dis. 2018 Dec 5;9(12):1178. doi: 10.1038/s41419-018-1212-7 (PMC6281581; doi:10.1038/s41419-018-1212-7)
Supplement: Supplementary file 3 — Supplementary methods [file 41419_2018_1212_MOESM3_ESM.docx]

**Supplementary methods**

**The construction of *ZEB1* heterozygous and homozygous knockout cell lines**

The analyses of *ZEB1* CN, mRNA/protein expression and DNA sequence in the available SS cell lines prompted us to use H9, showing a relatively high ZEB1 endogenous level, as the parental population to conduct a Non-Homologous End Joining (NHEJ) CRISPR/Cas9 mediated *ZEB1* knockout (KO) (**Figure S1**). We succeeded in isolating trans-edited H9 clones in heterozygous KO state according to the Fractional Allele abundance of the drop-off assay used as screening method; the clonal selection and mutational screening was performed twice and positive clones were verified by sequencing of the NHEJ target region. The clone designated as B8*ZEB1*^+/-^ carried a WT allele and a mutated c.1353delA allele causing a frame-shift in the protein reading frame which introduces a premature stop codon after 491 amino acid (aa) (**Figure S2**). The Western Blot (WB) analysis demonstrated a reduced ZEB1 expression in B8*ZEB1*^+/-^ cells and no band corresponding to a truncated protein was detected (**Figure S3**). The same CRISPR/Cas9 vector was then introduced in the heterozygous B8 KO clone and screened for homozygous KO. One positive clone, designated C9*ZEB1*^-/-^, was successfully isolated and sequence verified. Besides the previously described c.1353delA allele, this clone carried the second allele with c.1353insA mutation causing the insertion of a stop codon after 465 aa (**Figure S2**). The bi-allelic inactivation of *ZEB1* gene in C9*ZEB1*^-/-^ clone was verified by WB which showed neither specific band corresponding to ZEB1 nor other lower molecular weight bands of truncated proteins (**Figure S3**). The resulting *ZEB1* frame-shift mutations lead to a significant decrease in protein abundance compatible with the nonsense mediated decay effect of a gene KO (Popp M.W., Maquat L.E. Leveraging Rules of Nonsense-Mediated mRNA Decay for Genome Engineering and Personalized Medicine. Cell. 2016 Jun 2;165(6):1319-1322).
