# Supplementary material for: Loss of the candidate tumor suppressor ZEB1 (TCF8, ZFHX1A) in Sézary syndrome
Source: Cell Death Dis. 2018 Dec 5;9(12):1178. doi: 10.1038/s41419-018-1212-7 (PMC6281581; doi:10.1038/s41419-018-1212-7)
Supplement: Supplementary file 4 — Figure S1 [file 41419_2018_1212_MOESM4_ESM.pptx]

## Slide 1
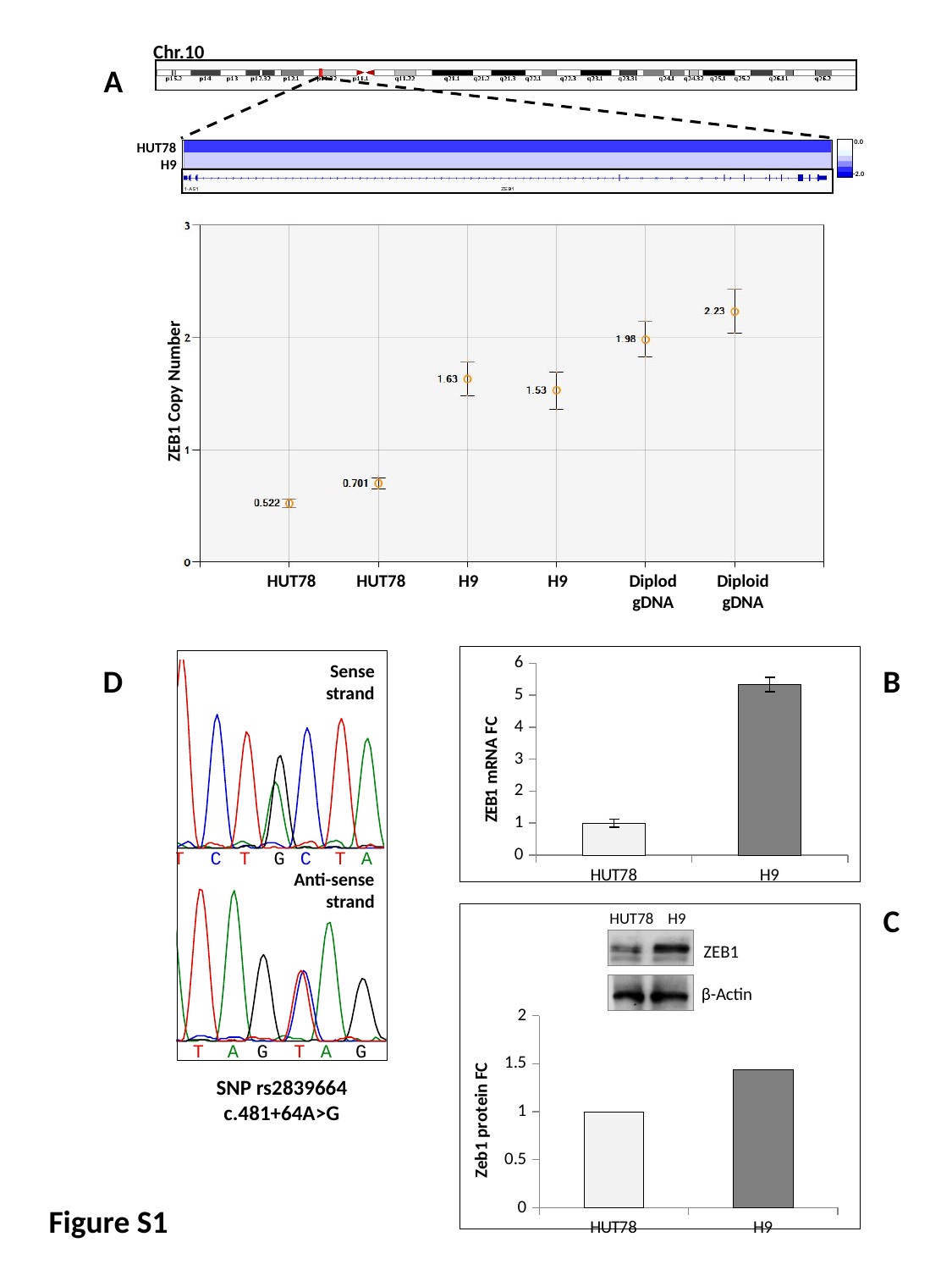

Chr.10
HUT78
H9
A
0.0
-2.0
ZEB1 Copy Number
HUT78
HUT78
H9
H9
Diplod
gDNA
Diploid
gDNA
### Chart
| Category | ZEB1 FC values |
|---|---|
| HUT78 | 1.0 |
| H9 | 5.333194708364777 |
Sense
strand
D
B
Anti-sense
strand
C
HUT78 H9
ZEB1
β-Actin
### Chart
| Category | ZEB1 |
|---|---|
| HUT78 | 1.0 |
| H9 | 1.4338536259426902 |SNP rs2839664
c.481+64A>G
Figure S1
