# Supplementary material for: Loss of the candidate tumor suppressor ZEB1 (TCF8, ZFHX1A) in Sézary syndrome
Source: Cell Death Dis. 2018 Dec 5;9(12):1178. doi: 10.1038/s41419-018-1212-7 (PMC6281581; doi:10.1038/s41419-018-1212-7)
Supplement: Supplementary file 5 — Figure S2 [file 41419_2018_1212_MOESM5_ESM.pptx]

## Slide 1
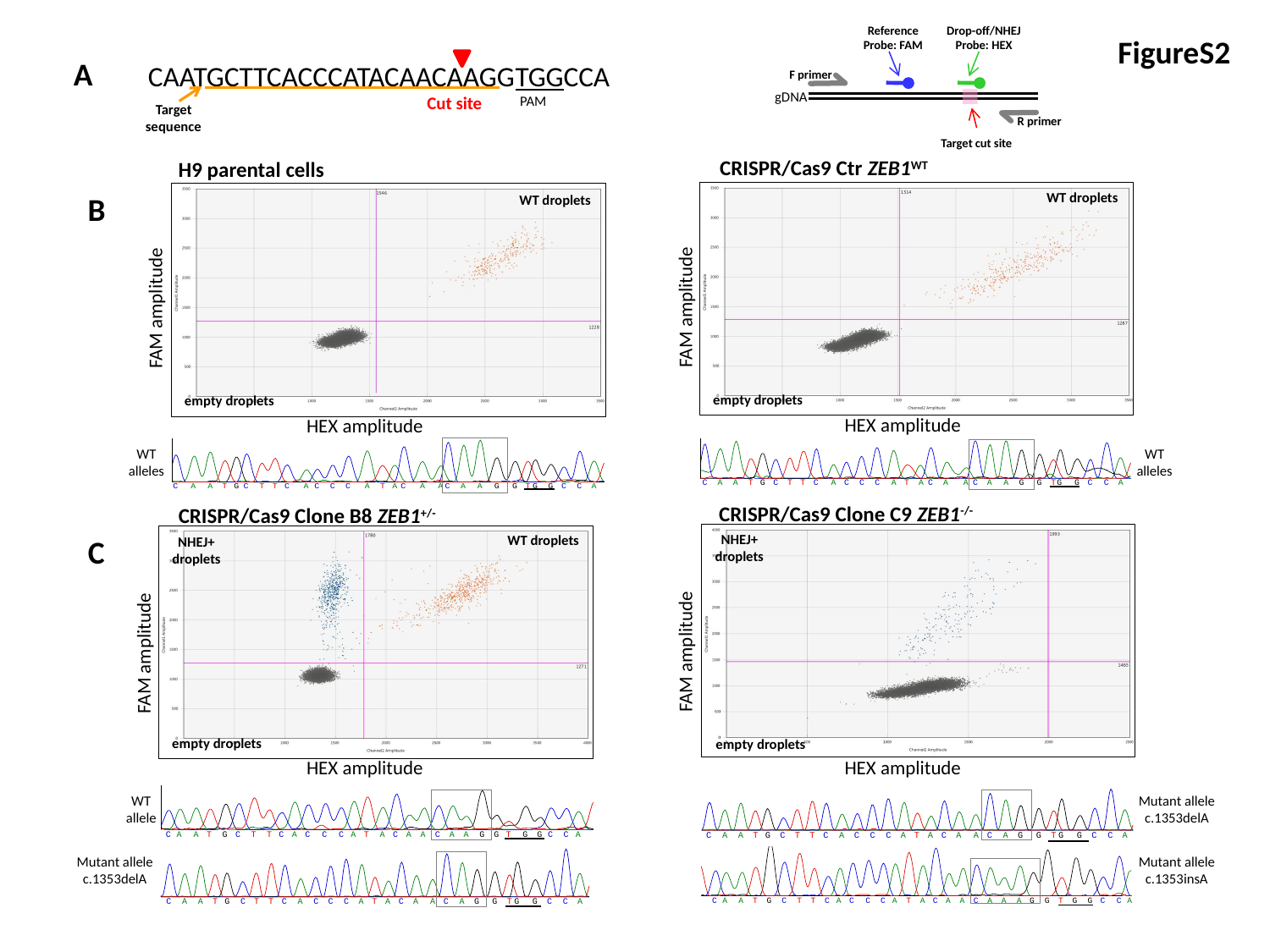

Reference
Probe: FAM
Drop-off/NHEJ
Probe: HEX
F primer
gDNA
R primer
Target cut site
FigureS2
A
CAATGCTTCACCCATACAACAAGGTGGCCA
PAM
Cut site
Target
sequence
CRISPR/Cas9 Ctr ZEB1WT
H9 parental cells
WT droplets
B
WT droplets
FAM amplitude
FAM amplitude
empty droplets
empty droplets
HEX amplitude
HEX amplitude
WT
alleles
WT
alleles
CRISPR/Cas9 Clone C9 ZEB1-/-
CRISPR/Cas9 Clone B8 ZEB1+/-
NHEJ+
droplets
WT droplets
C
NHEJ+
droplets
FAM amplitude
FAM amplitude
empty droplets
empty droplets
HEX amplitude
HEX amplitude
WT
allele
Mutant allele
c.1353delA
Mutant allele
c.1353delA
Mutant allele
c.1353insA
