# Supplementary figures and images for: Loss of the candidate tumor suppressor ZEB1 (TCF8, ZFHX1A) in Sézary syndrome
Source: Cell Death Dis. 2018 Dec 5;9(12):1178. doi: 10.1038/s41419-018-1212-7 (PMC6281581; doi:10.1038/s41419-018-1212-7)

## Slide 1
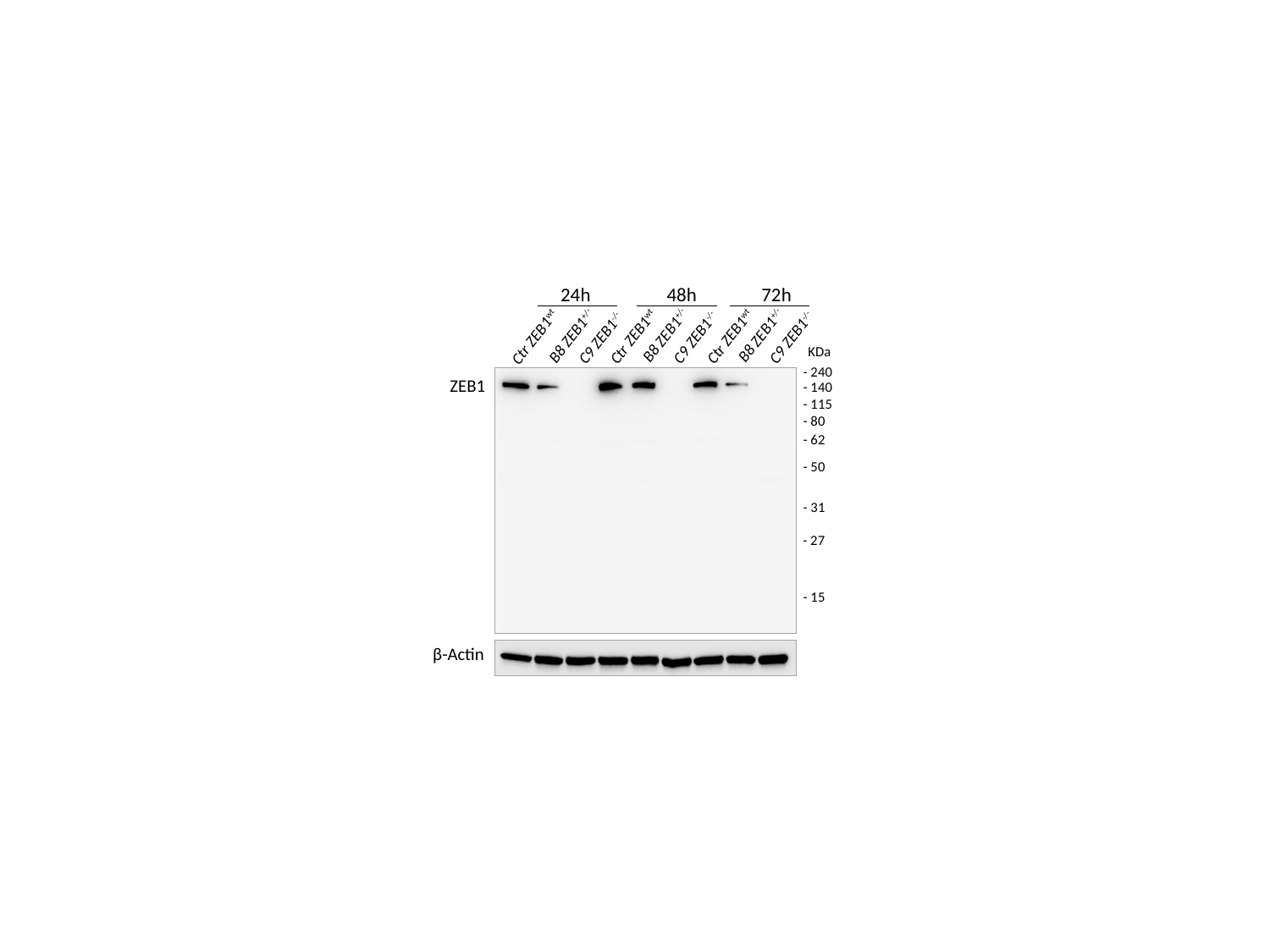

24h
48h
72h
B8 ZEB1+/-
B8 ZEB1+/-
B8 ZEB1+/-
Ctr ZEB1wt
Ctr ZEB1wt
Ctr ZEB1wt
C9 ZEB1-/-
C9 ZEB1-/-
C9 ZEB1-/-
KDa
- 240
- 140
- 115
- 80
- 62
- 50
- 31
- 27
- 15
ZEB1
β-Actin

Supplement: Supplementary file 6 — Figure S3 [file 41419_2018_1212_MOESM6_ESM.pptx]

## Slide 1
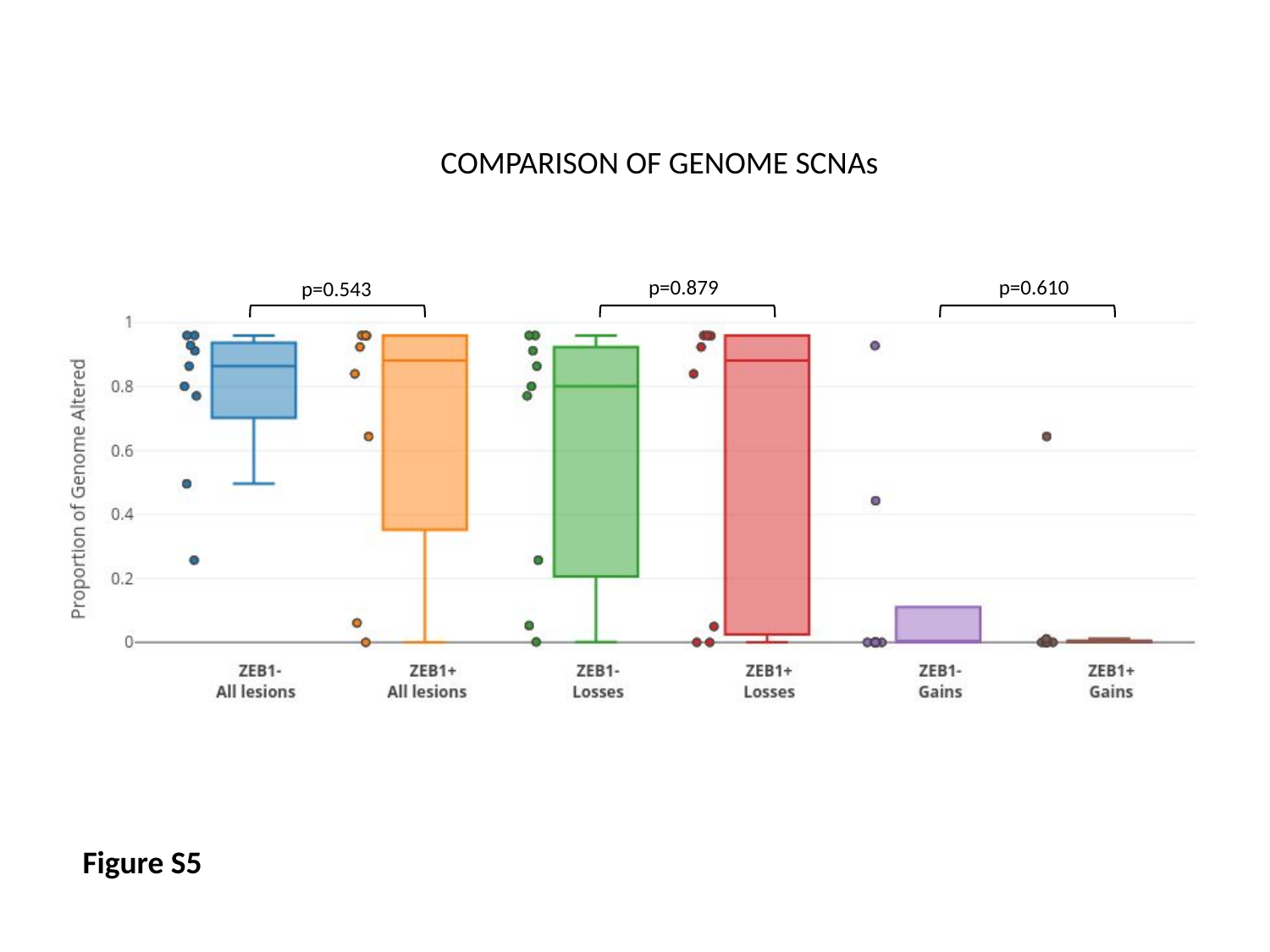

COMPARISON OF GENOME SCNAs
p=0.879
p=0.610
p=0.543
Figure S5

Supplement: Supplementary file 9 — Figure S5 [file 41419_2018_1212_MOESM9_ESM.pptx]
