# Supplementary material for: Loss of the candidate tumor suppressor ZEB1 (TCF8, ZFHX1A) in Sézary syndrome
Source: Cell Death Dis. 2018 Dec 5;9(12):1178. doi: 10.1038/s41419-018-1212-7 (PMC6281581; doi:10.1038/s41419-018-1212-7)
Supplement: Supplementary file 8 — Figure S4 [file 41419_2018_1212_MOESM8_ESM.pptx]

## Slide 1
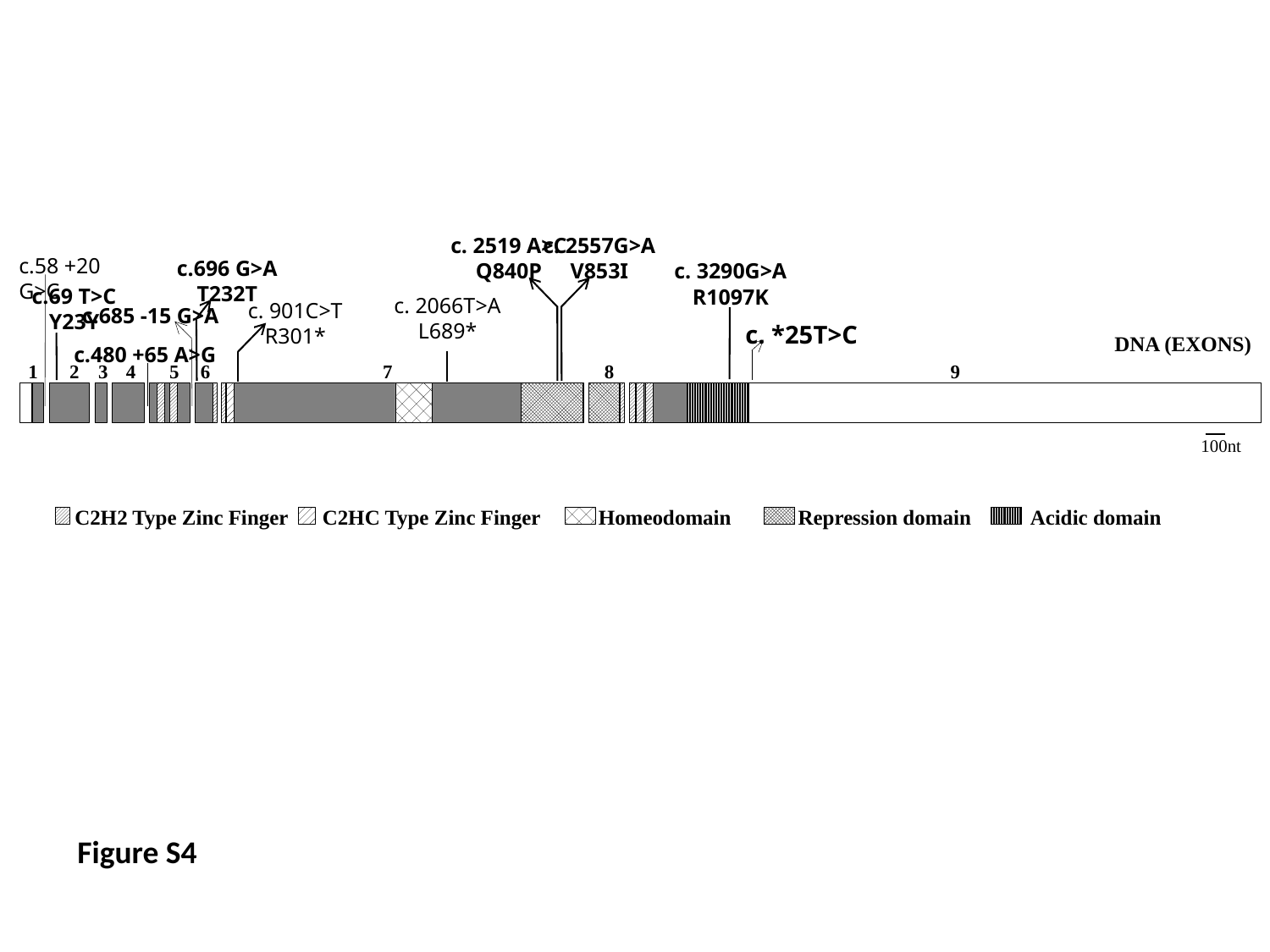

c. 2519 A>C
Q840P
c. 2557G>A
V853I
c.58 +20 G>C
c.696 G>A
T232T
c. 3290G>A
R1097K
c.69 T>C
Y23Y
c. 2066T>A
L689*
c. 901C>T
R301*
c.685 -15 G>A
c. *25T>C
DNA (EXONS)
c.480 +65 A>G
1
2
3
4
5
6
7
8
9
100nt
C2H2 Type Zinc Finger
C2HC Type Zinc Finger
Homeodomain
Repression domain
Acidic domain
Figure S4
