# Supplementary material for: Loss of the candidate tumor suppressor ZEB1 (TCF8, ZFHX1A) in Sézary syndrome
Source: Cell Death Dis. 2018 Dec 5;9(12):1178. doi: 10.1038/s41419-018-1212-7 (PMC6281581; doi:10.1038/s41419-018-1212-7)
Supplement: Supplementary file 10 — List of supplementary information [file 41419_2018_1212_MOESM10_ESM.docx]

LIST OF SUPPLEMENTARY INFORMATION FILES

**Table S1 Clinical features of SS patients analysed** listing the clinical characteristics of Sézary syndrome patients and references reporting further details. (xlsx 14KB)

**Table S2** listing the 12 couples of primers used for sequencing of *ZEB1* exons. (xlsx 11KB)

**Supplementary Methods.** The construction of *ZEB1* heterozygous and homozygous knockout cell lines. It describes the procedure employed to generate *ZEB1* KO cell lines, the screening method and the analyses of the successfully generated cell clones. (doc 12KB)

**Figure S1. A,** *ZEB1* copy number detected in the HUT78 and H9 parental cell lines by SNP6.0 array (upper panel, blue color grade denotes deletion amplitude) and validated by Droplet Digital PCR (ddPCR) (bottom panel), two reference probes were used for normalization and Affymetrix reference 103 for diploid genomic DNA control. **B** and **C**, real-time PCR and western blot analysis showing higher ZEB1 level in H9 cell line compared to HUT78. **D**, electropherogram of rs2839664 A>G polymorphism illustrating the presence of both alleles in H9 cell line. (pptx 135.9KB)

**Figure S2.** **A**, Left, the target sequence of the *ZEB1* gene, the PAM sequence is underlined and the predicted cut site is indicated. Right, primer and probe design for the Non-Homologous End Joining (NHEJ) drop-off assay. **B**, Left, ddPCR 2-D plot of H9 parental population genomic DNA (gDNA) showing only wt allele positive droplets due to the binding of both the reference blu (FAM) and the drop-off green (HEX) probes, the target sequence analysis is also displayed. Right, 2-D plot of the H9 cells transfected with the scrambled-gRNA carrying CRIPR/Cas9 plasmid (control) exhibiting the same results as the parental cells. **C**, Heterozygous knockout (KO) clone showing the presence of the recombinant mutant allele (FAM positive droplets indicate the binding of only the reference probe) and wt allele. The sequence analysis confirming the predicted mutation c.1353 del A in heterozygous state is shown. Right, ddPCR plot of the homozygous KO clone displaying only NHEJ positive droplets; the target sequences disclosed the c.1353 del A combined with the c.1353 ins A allele generating the *ZEB1* bi-allelic mutation. (pptx 342.5KB)

**Figure S3.** Western Blot analysis of ZEB1 showing the presence of the protein in the cells transfected with control CRISPR/Cas9 vector (Ctr *ZEB1*^wt^) and in the B8 *ZEB1*^+/-^ clone. No band corresponding to ZEB1 is present in the C9 *ZEB1*^-/-^ at any time of cell culture examined. No specific bands corresponding to truncating ZEB1 forms are visible in the B8 *ZEB1*^+/-^ or C9 *ZEB1*^-/-^ lanes. Due to the higher background of cell clones, dilutions of primary and secondary antibodies were increased to 1:500 and 1:25.000, respectively and immunodetection was performed with Clarity Western ECL substrate, ChemiDoc MP Image system and Image Lab software (Bio-Rad Laboratories, Hercules, CA). (pptx 378KB)

**Table S3** The significant regions of chromosomal changes identified by GISTIC in 24 SS tumors hybridized on SNP6 array. (xlsx 19KB)

**Figure S4** . *ZEB1* exons and location of all identified substitutions relative to the known protein structural and functional domains. Dark grey indicates the coding segments of DNA (introns length are not shown) while untranslated regions are in white. Protein domains are indicated in the legend. Bold font denotes germline variations/mutations. Mutation nomenclature refers to the coding DNA reference sequence (NCBI Refseq NM_030751) and follows the recommendations of the Human Genome Variation Society (den Dunnen, J.T. and Antonarakis, S.E. (2000). Hum.Mutat. 15: 7-12). (pptx 59.5KB)

**Figure S5.** Comparison of genome SCNAs. Comparison of 10K array segmented data from 9 SS samples with *ZEB1* homo-deletions/strong mRNA underexpression indicated as ZEB1- (P05T, P15T, P22T, P23T3, P25T, P27T, P28T, P32T1, P55T1 in Table 1) and 8 SS tumors with *ZEB1* positive expression, ZEB1+ (P30T1, P33T, P35T, P36T, P39T, P45T1, P49T1, P55T1 in Table 1). To quantitatively assess the distribution of SCNAs in the two groups of patients, we calculated the fraction of each tumor genome that was affected by SCNAs representing the Proportion of Genome Altered, as described by Thu KL et al. (36). No significant results were obtained considering all lesions (i.e. gains and losses), only losses or only gains using a two-tailed Student’s t-test assuming unequal variance (Welch's unpaired *t* test); p-values are shown above each comparison. Box plot were generated at <https://plot.ly/create/box-plot/#/> (pptx 88KB).
